# Efficacy of a Timed Activity Intervention to Improve Sleep: Findings From the Healthy Patterns Study

**DOI:** 10.1093/geroni/igab046.2091

**Published:** 2021-12-17

**Authors:** Nancy Hodgson, Miranda McPhillips, Darina Petrovsky, Subhash Aryal

**Affiliations:** 1 University of Pennsylvania, School of Nursing, philadelphia, Pennsylvania, United States; 2 University of Pennsylvania, University of Pennsylvania, Pennsylvania, United States; 3 Rutgers University, Philadelphia, Pennsylvania, United States; 4 University of Pennsylvania School of Nursing, Philadelphia, Pennsylvania, United States

## Abstract

We conducted a two-arm RCT with dyads of 200 persons living at home with dementia (PLWD) who reported sleep disruption and family caregivers. Components of the Healthy Patterns intervention included: 1) assessing PLWD functional status, preferences and interests; 2) educating caregivers on environmental cues to promote activity and sleep; and 3) training caregivers in timed morning, afternoon, and evening activities. Outcomes included: PLWD quality of life, sleep, and neuropsychiatric symptoms. Sleep-wake patterns were assessed using wrist actigraphy and proxy-reported measures. The main intervention effects were tested using ANCOVA. The average age of participants was 73.4 years, 67% were female, 80% were African American/Black). At 4 weeks, the intervention group demonstrated less sleep-related impairment (p = 0.0031) and reported higher quality of life than the control group (p = 0.0074). These results provide new fundamental knowledge regarding the effects of timing activity on sleep and well-being.

